# Three-Year Analysis of the Rectal Cancer Care Trajectory after the COVID-19 Pandemic

**DOI:** 10.3390/diseases11040181

**Published:** 2023-12-11

**Authors:** Vlad Braicu, Lazar Fulger, Aditya Nelluri, Ram Kiran Maganti, Uday Shree Akkala Shetty, Gabriel Verdes, Dan Brebu, Catalin Dumitru, Ana-Olivia Toma, Ovidiu Rosca, Ciprian Duta

**Affiliations:** 1 Doctoral School, “Victor Babes” University of Medicine and Pharmacy Timisoara, Eftimie Murgu Square 2, 300041 Timisoara, Romania; braicu.vlad@umft.ro (V.B.); ciprian_duta@yahoo.com (C.D.); 2 Department of General Surgery, “Victor Babes” University of Medicine and Pharmacy Timisoara, Eftimie Murgu Square 2, 300041 Timisoara, Romania; lazarfulger@yahoo.com (L.F.); gabriel.verdes@gmail.com (G.V.); dr.brebudan@gmail.com (D.B.); 3 School of General Medicine, Sri Siddhartha Medical College, Tumakuru 572107, India; nelluriaditya@gmail.com; 4 School of General Medicine, Sri Devaraj Urs Academy of Higher Education and Research, Kolar 563101, India; ramkiran.maganti11@gmail.com; 5 Malla Reddy Institute of Medical Sciences, Suraram Main Road 138, Hyderabad 500055, India; udayshree98@gmail.com; 6 Department of Obstetrics and Gynecology, “Victor Babes” University of Medicine and Pharmacy Timisoara, Eftimie Murgu Square 2, 300041 Timisoara, Romania; dumcatal@yahoo.com; 7 Department of Dermatology, “Victor Babes” University of Medicine and Pharmacy Timisoara, Eftimie Murgu Square 2, 300041 Timisoara, Romania; toma.olivia@umft.ro; 8 Department of Infectious Diseases, “Victor Babes” University of Medicine and Pharmacy Timisoara, Eftimie Murgu Square 2, 300041 Timisoara, Romania

**Keywords:** COVID-19, rectal cancer, surgical oncology

## Abstract

The global pandemic period from 2020 to 2022 caused important alterations in oncology care. This study aimed to describe the trends and variations in patient characteristics, comorbidities, and treatment approaches during this time in Romania. We conducted a retrospective database search to identify patients with rectal cancer who underwent surgical intervention between 2020 and 2022 and the year 2019, which served as a pre-pandemic period control. This study included 164 patients, with a yearly increase of approximately 10% in surgical interventions noted from 2020 (1709 interventions) to 2022 (2118 interventions), but an overall 34.4% decrease compared with the pre-pandemic period. Notable shifts were observed in the type of surgeries performed, with laparoscopic procedures doubling from 2020 (25%) to 2022 (47.5%), confirming the decrease in emergency presentations during the last year of the COVID-19 pandemic and a recovery to normality with planned, elective interventions. Elective interventions increased significantly in 2022 (79.7%) compared with the previous years (*p* = 0.043), with a concurrent rise in neoadjuvant therapy uptake in 2022 (35.6%). However, significant alterations in the TNM staging, from 12.5% stage IV cases in 2020 to 25.4% in 2022 (*p* = 0.039), indicated an increased diagnosis of advanced stages of rectal cancer as the years progressed. There was a significant difference in albumin levels over the years (*p* = 0.019) and in the American Society of Anesthesiology (ASA) scores (from 6.2% ASA stage IV in 2020 to 16.9% in 2022), denoting an increase in case complexity (*p* = 0.043). This study reveals a trend of increasing surgical interventions and the prevalence of more advanced stages of rectal cancer during the pandemic years. Despite the subtle fluctuations in various patient characteristics and treatment approaches, notable shifts were documented in the severity at diagnosis and surgery types, pointing toward more advanced disease presentations and changes in surgical strategies over the period studied. Nevertheless, the trends in ICU admission rates and mortality did not alter significantly during the pandemic period.

## 1. Introduction

Rectal cancer, considered one of the primary malignancies affecting the lower gastrointestinal tract, remains a substantial contributor to global cancer morbidity and mortality, with an estimated 700,000 new cases and 300,000 deaths reported globally in 2020 [1,2]. This malignancy necessitates a nuanced approach to management, often involving comprehensive strategies that integrate surgical interventions with radiation and systemic therapies [3,4,5]. Advanced rectal cancer stages require particularly intricate surgical techniques, including total mesorectal excision, which serves as a cornerstone in preventing local recurrence and ensuring optimal survival outcomes [6,7]. Furthermore, molecular advancements have allowed for a tailored approach to management, with various molecular subtypes of rectal cancer necessitating specific therapeutic pathways [8,9,10].

The COVID-19 pandemic, which emerged in early 2020, exerted immense pressure on healthcare systems across the globe, causing a noticeable shift in resource allocation and healthcare delivery patterns [11,12]. As healthcare facilities pivoted to address the burgeoning demands of the pandemic, the repercussions were felt intensely in the field of oncology [13,14]. Reports indicate a substantial decrease in the number of cancer diagnoses in the initial phase of the pandemic, with a study revealing an almost 50% reduction in weekly cancer presentations as compared with pre-pandemic figures [15]. This reduction was attributed largely to delays in screening and diagnostic services, raising concerns about potential increases in advanced-stage diagnoses in the subsequent years [16].

Moreover, the surgical management of rectal cancer encountered a significant disruption during the early stages of the pandemic [17]. A worldwide survey conducted among colorectal surgeons reported a staggering 85% decrease in elective surgeries, with an accentuated emphasis on emergency interventions to manage complications such as obstructions or perforations [18,19]. Furthermore, the pandemic necessitated rapid adaptations in treatment protocols, with increased adoption of short-course radiotherapy and modifications in chemotherapy regimens to reduce hospital visits and lower the risk of COVID-19 exposure among the vulnerable oncologic patient population.

In 2021, healthcare systems strived to recalibrate and adapt to the evolving dynamics of the pandemic, with efforts to reinstate standard oncologic care alongside managing the persistent threat of COVID-19 [20,21,22]. Telemedicine emerged as a viable alternative, facilitating remote consultations and reducing the necessity for physical visits to healthcare facilities [23]. However, the decrease in face-to-face consultations raised concerns regarding potential delays in identifying disease progression or complications, thereby accentuating the need for a careful evaluation of the new care delivery models. Thus, by 2022, it became clear that the COVID-19 pandemic had a significant impact on surgical oncology, leaving the questions of when everything will go back to what was considered normality before the pandemic and what are the short-term and long-term consequences [24,25].

This study hypothesizes that the management and surgical outcomes of rectal cancer patients returned to pre-pandemic normality in 2022, following the disruptions caused by the pandemic restrictions in 2020 and 2021. The primary objective of this research was to perform a comparative analysis of patient outcomes, focusing on treatment delays, surgical outcomes, and survival rates across the years 2020, 2021, and 2022 in comparison with the pre-pandemic period of 2019.

## 2. Materials and Methods

### 2.1. Study Design and Ethics

This research used a retrospective design based on a three-year observational study that took place at the Timiș County Emergency Clinical Hospital. The study spanned the pandemic years 2020 to 2022, comprising the trajectory of rectal cancer care during and after the COVID-19 pandemic, in comparison with the pre-pandemic year of 2019. The period starting in March 2020, when the COVID-19 pandemic was officially announced and quarantine restrictions began, was defined as the COVID-19 pandemic phase. The study protocol underwent a comprehensive evaluation and subsequent approval by the hospital’s Institutional Review Board on 6 June 2022, with approval number 304, and strictly adhered to the Declaration of Helsinki guidelines. Every effort was made to ensure that the participants were well-informed of the potential risks and benefits associated with the study, fostering an environment of trust and open communication.

### 2.2. Inclusion and Exclusion Criteria

Our study specifically encompasses patients who were diagnosed and underwent treatment for rectal cancer at the Timiș County Emergency Clinical Hospital during the years 2019 to 2022. This time frame was chosen to provide an insightful comparison of rectal cancer care during the peak of the COVID-19 pandemic and in the subsequent period when healthcare systems were in the recovery phase, as well as a comparison with the pre-pandemic period. It was necessary to include patients’ medical records documenting their treatment journey, encompassing details such as surgery specifics, radiotherapy schedules, and the overall response to the administered treatments. Moreover, participants included in this study were individuals who expressed a willingness to participate in research studies, thereby providing informed consent that permits researchers to analyze their medical data while ensuring the confidentiality of their personal information.

On the other hand, the exclusion criteria comprised patients with colorectal cancer identified in a different segment of the large intestine other than the rectum. Patients with incomplete medical records, or those who were diagnosed with rectal cancer before the onset of the COVID-19 pandemic were excluded. Additionally, patients who were unable or unwilling to adhere to the study protocol or those who could not provide informed consent were not considered for inclusion.

### 2.3. Data Collection and Study Variables

The process of data collection included a wide array of variables with an emphasis on capturing detailed patient data for a thorough understanding of the recovery trajectory during the specified years, comprising demographic details, clinical history, treatment regimens, and specific medical parameters linked to rectal cancer care.

These variables encompassed an extensive range of parameters including the following: age, gender, height, weight, Body Mass Index (BMI), abdominal circumference, SM Index, visceral obesity, ASA score, radiotherapy history for other neoplasia, comorbidities, neoplastic history, hemoglobin, hematocrit, total proteins, albumin, TNM staging, distance from the anal verge, MRI location, neoadjuvant therapy and its type, radiation therapy dates if applicable (first and last session), operation date, approach and conversion in surgery, intraoperative complications, tumor localization and properties (location, anatomic relations, circular extent, etc.), postoperative management and outcome (management, Clavien–Dindo classification, ICU stay, discharge date, etc.), and mortality.

### 2.4. Statistical Analysis

Data management and analysis were conducted utilizing the statistical software SPSS version 26.0 (SPSS Inc., Chicago, IL, USA). Continuous variables were represented as mean ± standard deviation (SD), while categorical variables were expressed in terms of frequencies and percentages. To analyze the changes between more than two means of continuous variables, the ANOVA test was utilized. The Chi-square test was utilized for the categorical variables. A *p*-value threshold of less than 0.05 was set for statistical significance.

## 3. Results

**Figure 1:**
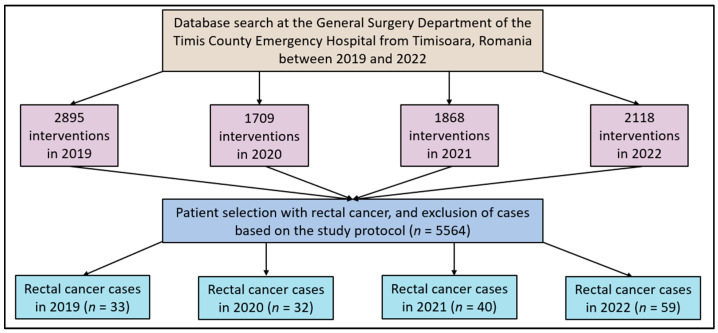
Study flowchart.

The retrospective database search identified a total of 131 patients with rectal cancer during the three-year pandemic period who were eligible for inclusion in the final analysis, as well as 33 patients identified in the year 2019, before the COVID-19 pandemic. A total of 32 cases of rectal cancer were identified in 2020 out of 1709 surgical interventions (1.9%), 40 in 2021 out of 1868 interventions (2.1%), and 59 in 2022 out of a total number of 2118 surgical interventions (2.8%). In comparison, in the pre-pandemic period, there were significantly more interventions, with a total of 2895, and a lower proportion of rectal cancer cases (1.1%). Even though there was no statistically significant difference between the three pandemic years (*p*-value = 0.147), there was an approximately 10% yearly increase in the total number of surgical interventions, from 1709 in 2020 to 2118 in 2022, but an overall significant decrease in total interventions as compared with the pre-pandemic year 2019, as presented in Figure 1.

Several significant trends were noted regarding the characteristics of patients diagnosed with rectal cancer from 2020 to 2022, as outlined in Table 1. Across the three pandemic years, the average age of patients at the time of diagnosis exhibited slight fluctuations, with the mean ages being 66.2, 64.0, and 67.4 for the years 2020, 2021, and 2022, respectively. The statistical analysis demonstrated that these variations were not significant (*p* = 0.324), implying a steady age distribution among the patients throughout the observed period.

Regarding the gender distribution, a slightly higher proportion of male patients were documented in 2020 and 2022, constituting 62.5% and 59.3% of the diagnosed cases, respectively, compared with 52.5% in 2021. However, the differences were not statistically significant (*p* = 0.668). The Body Mass Index (BMI) classifications revealed that the majority of the patients were either overweight or obese across all three years, showing a mild increase in the prevalence of obesity, from 34.4% in 2020 to 42.4% in 2022 (*p* = 0.760).

In terms of the Skeletal Muscle Index (SMI), this study found a slight decline in values for males and a relatively inconsistent pattern for females over the years. However, these variations did not represent a significant trend. Further, this study noted a moderate prevalence of visceral obesity across the three years, with no significant variations (*p* = 0.803).

When analyzing the comorbidities, cardiovascular diseases were found to be the most prevalent among the patients, although a decreasing trend was noted from 62.5% in 2020 to 49.2% in 2022 (*p* = 0.308). Other comorbidities, including pulmonary diseases, diabetes mellitus, cerebrovascular conditions, and renal disease, remained relatively stable over the years, with no significant differences in their prevalence rates. Regarding previous histories of neoplasia, the data showed a decreasing trend, dropping from 9.4% in 2020 to 3.4% in 2022 (*p* = 0.476). Moreover, the utilization of steroids or immunotherapy within the past three months before diagnosis was documented to be minimal throughout the years, with no significant variation (*p* = 0.507).

Throughout the three-year period, subtle fluctuations in preoperative laboratory data were documented. The average hemoglobin levels depicted anemia in the majority of patients and minor variations, with values recorded at 10.3 in 2020, a slight decrease to 9.8 in 2021, and then a rise to 10.9 in 2022, without a significant difference (*p* = 0.323). Meanwhile, the hematocrit levels stayed constant with means ranging from 36.5 to 39.4, without notable statistical differences (*p* = 0.170). However, a significant difference was observed in albumin levels over the years (*p* = 0.019), with a slight decrease in 2021 (3.8 ± 0.8) compared with 2020 (4.0 ± 0.7), and a subsequent increase in 2022 (4.2 ± 0.6). The total protein levels demonstrated a consistent trend with a slight increase in 2022; however, without a significant *p*-value (*p* = 0.109).

**Figure 2:**
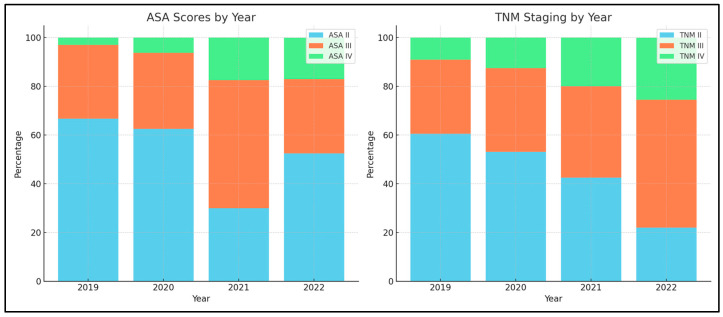
Comparison of ASA and TNM variations across the three pandemic years in comparison with the pre-pandemic year 2019.

A noteworthy variation was perceived in the American Society of Anesthesiology (ASA) scores, with a significant *p*-value of 0.043. The data revealed a prominent shift from a larger percentage of patients having an ASA score of II in 2020 (62.5%) to an increased prevalence of scores III and IV in the subsequent years, denoting a possible escalation in the complexity of cases over the period. The TNM staging also unveiled significant alterations over the pandemic years (*p* = 0.039), as presented in Figure 2. In particular, a notable decrement in stage II cases and an increase in stages III and IV were observed, indicating a trend in diagnosing more advanced stages of rectal cancer as the years progressed.

Regarding metastasis, a slight but non-significant increase in both local and distant metastases, as well as local invasions, were seen throughout the study period, with *p*-values of 0.244, 0.643, and 0.450, respectively. The metastasis location data illustrated a relatively stable distribution in the occurrence in the lungs, liver, and peritoneal regions, with the addition of other locations in 2022 (*p* = 0.867). Examining tumor distance from the anal verge, a marginal variation was observed over the three years (*p* = 0.646), indicating a relatively consistent pattern in tumor locations. Moreover, the position of the tumors based on MRI remained relatively constant, with a higher frequency of high-position tumors throughout the period (between 47.5% in 2021 and 61.0% in 2022), as described in Table 2.

The analysis of patient presentation exhibited a significant transformation over the three-year period. While emergency interventions were stable in 2020 and 2021, constituting 37.5% and 42.5% of cases, respectively, a marked decline was noticed in 2022, with only 20.3% of cases requiring emergency attention. Conversely, elective interventions were more common in 2022, reaching a total of 79.7% compared with 62.5% in 2020 and 57.5% in 2021. Neoadjuvant therapy uptake experienced a significant increase in 2022, with 35.6% of patients undergoing this therapy, as opposed to 18.8% and 15.0% in 2020 and 2021, respectively (*p* = 0.043).

**Figure 3:**
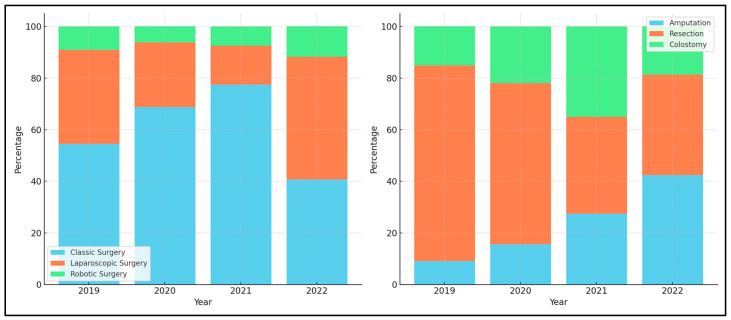
Comparison of surgical type and surgical method for rectal cancer during and before the COVID-19 pandemic.

A remarkable change was observed in the types of surgery performed (*p* = 0.004), as seen in Figure 3. The traditional classic surgery saw a decline from 68.8% in 2020 to 40.7% in 2022, with laparoscopic procedures almost doubling to 47.5% in 2022 from 25.0% in 2020. Furthermore, the utilization of robotic techniques saw a slight increase, though they remained a lesser-used option. The surgical methods showcased a significant difference over the period (*p* = 0.027), with amputations increasing noticeably from 15.6% in 2020 to 42.4% in 2022, and a corresponding decrease in resections from 62.5% to 39.0%.

The vascular ligation of the inferior mesenteric artery (IMA) and multiorgan resection displayed no significant variations over the years (*p* = 0.393 and *p* = 0.373, respectively), indicating stability in these surgical approaches. Similarly, the cases of splenic flexure mobilization were steady over the three years (*p* = 0.695). Anastomosis strategies remained largely consistent over the years in terms of types (*p* = 0.885) and methods utilized (*p* = 0.656), showcasing stability in surgical conclusions. The number of positive lymph nodes identified displayed an incremental trend but without a significant *p*-value (*p* = 0.219), indicating the need for vigilant lymph node assessments, as presented in Table 3.

The analysis demonstrated that the biological response to radiation therapy remained relatively stable across the three years, with 12.5% in 2020, 10.0% in 2021, and 11.9% in 2022, and no statistical significance was noted (*p* = 0.938). Similarly, there were no significant differences regarding Glasgow Coma Scale scores and signs of sepsis or post-operative complications, such as tachypnea, hypothermia, fever, systolic blood pressure, or the necessity for pressor support and the necessity for mechanical ventilation.

Concerning complications, peritoneal contamination showed a slight decreasing trend over the three years, though without a significant *p*-value (*p* = 0.828), indicating the need for continuous monitoring in subsequent years. The rates of ischemia at the resection margins remained low and consistent throughout the study period (<5%), indicating no significant change (*p* = 0.290). Reintervention rates also appeared to decrease marginally over the years without reaching statistical significance (*p* = 0.475).

When assessing the severity of complications using the Clavien–Dindo classification, the data illustrated that most patients experienced low to moderate scores (I to III) with a slight increase in scores I and II in 2022 (32.2% and 37.3%, respectively) compared with previous years. However, the overall distribution of scores did not indicate a significant variation (*p* = 0.704). Notably, ICU admission rates seemed to decrease from 15.6% in 2020 to 6.8% in 2022, though without reaching statistical significance (*p* = 0.314), potentially pointing toward an improvement in post-operative management strategies. Similarly, mortality rates showed a declining trend from 9.4% in 2020 to 3.4% in 2022, yet without statistical significance (*p* = 0.474), as presented in Table 4.

## 4. Discussion

In this three-year observational study delineating the recovery trajectory of rectal cancer care after the COVID-19 pandemic, several statistically significant transformations were noted. One area that necessitates further examination is the alterations in laboratory data across the years, particularly concerning the albumin levels. Our study witnessed a significant fluctuation in albumin levels over the years, which warrants a deeper investigation to decipher the underlying causes and their possible association with surgical outcomes, as it can be hypothesized that complications such as wound healing, scar formation, and development of fistulas may be influenced by albumin and total protein levels [26].

The COVID-19 pandemic period also witnessed significant alterations in the ASA score (*p* = 0.043) and TNM staging (*p* = 0.039), indicating an escalation in the severity of cases and a higher proportion of patients presenting with advanced stages of the disease during the pandemic. This shift toward more advanced disease presentations could be a consequence of delayed diagnoses and treatments due to healthcare disruptions during the pandemic. The management strategies also adapted significantly, evidenced by the increase in neoadjuvant therapy (*p* = 0.043) and laparoscopic surgeries (*p* = 0.004), reflecting a shift toward more conservative and minimally invasive approaches.

Regarding screening and delays in patient presentation, one meta-analysis pointed out that, in three out of seven scrutinized studies, postponing elective resections was associated with decreased overall or disease-free survival (DFS) rates [27]. The research noted that a one-month deferment in surgery was correlated with a 1.13 times increased chance of mortality, and a tripling of the delay escalated the risk to 1.57 times relative to the baseline. The estimated number of cases required to demonstrate detrimental effects was 35 for a one-month delay, which decreased to 10 for a three-month delay. The meta-analysis inferred that the delays had a non-significant adverse relationship with DFS, thus suggesting that colorectal cancer patients should avoid delaying elective surgeries beyond four weeks, as existing data indicate that extended delays post-diagnosis can result in suboptimal outcomes [28].

Studies from different countries and regions also described that the COVID-19 pandemic substantially reduced the diagnosis of new colorectal cancer cases worldwide. Countries like Spain and Brazil witnessed a decrease of almost 50% [29,30], with the UK observing reductions between 40% and 60% in various studies. Nevertheless, country-specific differences will continue to exist [31,32,33]. The UK reported a severe decrease in weekly detected cases by 70%, translating to an undiagnosed number of almost 3000 cases of colorectal cancer during the pandemic [34]. Furthermore, the number of monthly diagnoses fell from 44 cases (2017–2019 average) to 30 by February 2020. In Italy, a range of studies indicated a decrease of 11% to 46% in 2020 [35,36], whereas Hong Kong experienced a 37% decline, reducing weekly cases from 90 to approximately 60 [37]. Meanwhile, the Netherlands saw a significant drop, especially in individuals below 55 and above 75 during the early weeks of April 2020 [38]. The weekly decline in rectal cancer cases could not be performed in our study due to the small size of the clinic.

The pandemic not only impacted general diagnoses but also escalated emergency diagnoses and cases presented in advanced stages, as observed in our study and described in other European countries. In Spain, emergency diagnoses rose from 3.6% in 2019 to 12.1% in 2020 [29], a trend echoed in the UK, which saw an increase in T4-stage cancer diagnoses from 27.1% (2018–2019) to 34.5% in 2020. Additionally, the rate of colon obstructions doubled in the UK in 2020 compared with 2018–2019 [39]. Japan noted a surge in emergency admissions from 18.2% to 38.7% and a significant increase in colon obstructions [40]. Similarly, our study also identified more severe patient presentations as emergencies and cases that were too advanced and required stoma placement for bowel obstruction. Meanwhile, screening programs were severely impacted, with a substantial decrease in diagnoses through this route, as observed in countries like Spain and the Netherlands. Furthermore, the pandemic resulted in delayed diagnoses, with the UK witnessing an increase in delayed cases from 12% in 2019 to 26% in 2020 [34]. Consequently, the efficacy of diagnostic methods changed, with the UK and Hong Kong reporting changes in the number of procedures required to diagnose a single case and the rate of positive diagnoses per endoscopy, respectively.

With regard to surgical interventions, a substantial transformation was observed in the types of surgeries performed over the three years (*p* = 0.004). The decline in traditional classic surgeries coupled with the rise in laparoscopic procedures underscores a pivotal shift in surgical approaches, possibly leaning toward minimally invasive techniques. Nevertheless, this trend became normality in many developed countries a decade ago [41]. Moreover, the surgical methods depicted a significant shift (*p* = 0.027), with an increased reliance on rectal amputations in 2022. Understanding the underlying reasons for this increase is crucial in guiding future surgical planning, although this was likely attributed to the increased elevation in cancer staging observed during the pandemic years, since more patients presented as an emergency.

Even though our study reported a statistically significant increase in neoadjuvant therapy in 2022 (*p*-value = 0.043), it is questionable whether the difference remains significant with a bigger sample size. Various other studies reported that the COVID-19 pandemic has notably disrupted the treatment timelines for colorectal cancer, potentially jeopardizing patient survival outcomes [42]. A significant decline in referrals for various treatments including surgeries, chemotherapy, and long-term radiation therapy has been observed, with only emergency surgeries and short-term radiotherapy experiencing an increasing trend [34]. Interestingly, colorectal resection surgeries in Italy and chemotherapy cases in New Zealand remained steady before and after the onset of the pandemic [43,44]. Furthermore, the pandemic has protracted the waiting times for hospital admissions up to 16 weeks, with an increased duration of post-surgery hospital stays reported globally, barring the UK, where the average hospital stay shortened for patients undergoing surgery during this period compared with those who had sigmoidoscopies before the pandemic [34].

Further research across various regions has highlighted specific healthcare alterations during the pandemic period. A generalized reduction in the utilization of primary care services was observed, largely attributed to a lack of resources within healthcare systems [45,46,47]. This reduction was compounded by barriers both within the service provision and among the population. Predominant among these were fears of SARS-CoV-2 contagion and stigma, as well as socioeconomic and technological challenges, which led to unmet healthcare needs and widened inequalities in access [48]. These issues were particularly pronounced among people with disabilities in six countries, who faced additional hurdles such as the restructuring of healthcare services, economic burdens hindering affordability, and a lack of support for care-seeking. Interestingly, despite these challenges, the impact of COVID-19 fears on healthcare-seeking behavior was relatively limited in developing countries compared with developed countries, where the fear of adverse effects caused by the SARS-CoV-2 virus was higher. However, the pandemic also introduced new dynamics in healthcare governance, notably increasing the reliance on international donors for health planning and occasionally creating friction between national and international health factors [49].

One of the study limitations is the retrospective design, and drawing data from a single medical facility, which might limit the generalizability of the findings to broader contexts, as different healthcare systems might have experienced varied impacts and responses during the pandemic. Nevertheless, this methodology was previously reported in other studies [50]. Secondly, the exclusion of patients with incomplete medical records or those diagnosed before the COVID-19 pandemic may have potentially excluded a subset of individuals who could have offered additional insights into the long-term trends and outcomes in rectal cancer care. Furthermore, although this study documented a detailed range of variables, it could benefit from incorporating a qualitative component to identify nuanced perspectives of patients’ experiences throughout their treatment journey. Lastly, even though the first reported COVID-19 case in Romania was in February 2020, and the pandemic was officially declared by WHO in March 2020, we acknowledge the potential oversight in assuming that the entirety of 2020 was impacted by the pandemic. This assumption might not fully represent the varied onset and progression of COVID-19 across different regions and its differential impact throughout the year.

## 5. Conclusions

This study observed notable shifts in rectal cancer management throughout the 2020 to 2022 COVID-19 pandemic period compared with the 2019 pre-pandemic period, marked by significant transformations in surgical interventions, diagnostic stages, and clinical complexities. There was an increase in surgical interventions, predominantly characterized by a doubling in laparoscopic procedures, underlining a transition toward planned, elective interventions, especially in the year 2022. This transition was also mirrored in the increase in neoadjuvant therapy applications in the same year. Alarmingly, there was an important increase in the diagnoses of advanced stages of rectal cancer and a notable increase in case complexity, as evidenced by elevated ASA scores and altered albumin levels across the years under study. Despite these changes, it is reassuring to note the stability in ICU admission and mortality rates, which did not exhibit significant fluctuations during the observed period, although it is unclear in the long term.

## Figures and Tables

**Table 1:** Background characteristics of patients diagnosed with rectal cancer during the period 2020–2022.

Variables	Control—2019 (n = 33)	2020 (n = 32)	2021 (n = 40)	2022 (n = 59)	*p*-Value
Age (mean ± SD)	65.5 ± 9.8	66.2 ± 10.2	64.0 ± 10.8	67.4 ± 10.5	0.324
Sex (male)	21 (63.6%)	20 (62.5%)	21 (52.5%)	35 (59.3%)	0.668
BMI (kg/m^2^)					0.760
Normal weight (18.5–25)	9 (27.3%)	8 (25.0%)	7 (17.5%)	15 (25.4%)	
Overweight (25–30)	12 (36.4%)	13 (40.6%)	17 (42.5%)	19 (32.2%)	
Obese (>30)	12 (36.4%)	11 (34.4%)	16 (40.0%)	25 (42.4%)	
SMI					
Male	54.7 ± 6.4	55.8 ± 6.6	53.2 ± 6.0	53.9 ± 7.2	0.196
Female	42.0 ± 5.8	41.3 ± 5.9	42.8 ± 6.2	40.5 ± 4.0	0.130
Visceral obesity (n, %)	8 (24.2%)	9 (28.1%)	14 (35.0%)	20 (33.9%)	0.803
Comorbidities					
Cardiovascular	18 (54.5%)	20 (62.5%)	18 (45.0%)	29 (49.2%)	0.308
Pulmonary	4 (12.1%)	5 (15.6%)	5 (12.5%)	8 (13.6%)	0.927
Diabetes mellitus	8 (24.2%)	7 (21.9%)	9 (22.5%)	11 (18.6%)	0.878
Cerebrovascular	3 (9.1%)	3 (9.4%)	4 (10.0%)	8 (13.6%)	0.788
Renal disease	2 (6.1%)	3 (9.4%)	3 (7.5%)	6 (10.2%)	0.901
Previous history of neoplasia	3 (9.1%)	3 (9.4%)	2 (5.0%)	2 (3.4%)	0.476
Steroids or immunotherapy during the past 3 months	1 (3.0%)	1 (3.1%)	0 (0.0%)	2 (3.4%)	0.507

SD—standard deviation; BMI—Body Mass Index; SMI—Skeletal Muscle Index (less than 52.4 cm^2^/m^2^ for men and 38.5 cm^2^/m^2^ for women as indicative of sarcopenia).

**Table 2:** Medical and oncological characteristics of patients with rectal cancer during the period 2020–2022.

Variables	Control—2019 (n = 33)	2020 (n = 32)	2021 (n = 40)	2022 (n = 59)	*p*-Value
Preoperative laboratory data					
Hemoglobin	10.2 ± 3.2	10.3 ± 3.3	9.8 ± 3.5	10.9 ± 9.8	0.323
Hematocrit	38.5 ± 7.0	38.7 ± 7.1	36.5 ± 8.9	39.4 ± 6.8	0.170
Albumin	4.1 ± 0.6	4.0 ± 0.7	3.8 ± 0.8	4.2 ± 0.6	0.019
Total proteins	7.1 ± 0.8	7.0 ± 0.9	6.8 ± 1.1	7.2 ± 0.8	0.109
ASA score					0.043
II	22 (66.7%)	20 (62.5%)	12 (30.0%)	31 (52.5%)	
III	10 (30.3%)	10 (31.3%)	21 (52.5%)	18 (30.5%)	
IV	1 (3.0%)	2 (6.2%)	7 (17.5%)	10 (16.9%)	
TNM Staging					0.039
II (all subtypes)	20 (60.6%)	17 (53.1%)	17 (42.5%)	13 (22.0%)	
III (all subtypes)	10 (30.3%)	11 (34.4%)	15 (37.5%)	31 (52.5%)	
IV (all subtypes)	3 (9.1%)	4 (12.5%)	8 (20.0%)	15 (25.4%)	
Metastasis					
Local	2 (6.1%)	1 (3.1%)	2 (5.0%)	7 (11.9%)	0.244
Distant	3 (9.1%)	4 (12.5%)	7 (17.5%)	12 (20.3%)	0.643
Local invasion	3 (9.1%)	3 (9.4%)	3 (7.5%)	9 (15.3%)	0.450
Metastasis location					0.867
Lungs	2 (6.1%)	1 (3.1%)	1 (2.5%)	4 (6.6%)	
Liver	4 (12.1%)	5 (15.6%)	7 (17.5%)	10 (16.9%)	
Peritoneal	3 (9.1%)	4 (12.5%)	3 (7.5%)	7 (11.9%)	
Other	0 (0.0%)	0 (0.0%)	0 (0.0%)	2 (3.4%)	
Distance from the anal verge (mean ± SD)	12.5 ± 6.9	12.7 ± 7.1	11.9 ± 6.8	13.2 ± 6.6	0.646
Position based on MRI					0.626
Low	6 (18.2%)	5 (15.6%)	9 (22.5%)	12 (20.3%)	
Medium	10 (30.3%)	8 (25.0%)	12 (30.0%)	11 (18.6%)	
High	17 (51.5%)	19 (59.4%)	19 (47.5%)	36 (61.0%)	

ASA—American Society of Anesthesiology; TNM—tumor node metastasis; SD—standard deviation; MRI—magnetic resonance imaging.

**Table 3:** Management of patients with rectal cancer during the period 2020–2022 in comparison with the pre-pandemic year 2019.

Variables	Control—2019 (n = 33)	2020 (n = 32)	2021 (n = 40)	2022 (n = 59)	*p*-Value
Patient presentation					0.045
Emergency intervention	10 (30.3%)	12 (37.5%)	17 (42.5%)	12 (20.3%)	
Elective surgery	23 (69.7%)	20 (62.5%)	23 (57.5%)	47 (79.7%)	
Neoadjuvant therapy	12 (36.4%)	6 (18.8%)	6 (15.0%)	21 (35.6%)	0.043
Type of neoadjuvant therapy					0.456
Chemo-radiotherapy	5 (15.2%)	1 (3.1%)	3 (7.5%)	11 (18.6%)	
Short course following chemotherapy	4 (12.1%)	3 (9.4%)	1 (2.5%)	7 (11.9%)	
Chemotherapy following short course	3 (9.1%)	2 (6.3%)	2 (5.0%)	3 (5.1%)	
Preoperative colon preparation					0.031
No preparation	8 (24.2%)	12 (37.5%)	17 (42.5%)	12 (20.3%)	
Laxative	16 (48.5%)	13 (40.6%)	10 (25.0%)	33 (55.9%)	
Enema	9 (27.3%)	7 (21.9%)	13 (32.5%)	14 (23.7%)	
Presence of stoma	2 (6.1%)	1 (3.1%)	1 (2.5%)	3 (5.1%)	0.783
Type of surgery					0.004
Classic	18 (54.5%)	22 (68.8%)	31 (77.5%)	24 (40.7%)	
Laparoscopy	12 (36.4%)	8 (25.0%)	6 (15.0%)	28 (47.5%)	
Robotic	3 (9.1%)	2 (6.3%)	3 (7.5%)	7 (11.9%)	
Surgical method					0.027
Amputation	3 (9.1%)	5 (15.6%)	11 (27.5%)	25 (42.4%)	
Resection	25 (75.8%)	20 (62.5%)	15 (37.5%)	23 (39.0%)	
Colostomy	5 (15.2%)	7 (21.9%)	14 (35.0%)	11 (18.6%)	
Surgical conversion	2 (6.1%)	3 (9.4%)	4 (10.0%)	9 (15.3%)	0.627
Vascular ligation of the IMA					0.393
High tie	13 (39.4%)	12 (37.5%)	24 (60.0%)	31 (52.5%)	
Low tie	16 (48.5%)	15 (46.9%)	12 (30.0%)	19 (32.2%)	
Unknown	4 (12.1%)	5 (15.6%)	4 (10.0%)	9 (15.3%)	
Splenic flexure mobilization	10 (30.3%)	9 (28.1%)	15 (37.5%)	19 (32.2%)	0.695
Multiorgan resection	3 (9.1%)	2 (6.3%)	5 (12.5%)	3 (5.1%)	0.373
Type of anastomosis					0.885
End to end	31 (93.9%)	30 (93.8%)	37 (92.5%)	56 (94.9%)	
End to side	2 (6.1%)	2 (6.3%)	3 (7.5%)	3 (5.1%)	
Method of anastomosis					0.656
Double stapled	24 (72.7%)	23 (71.9%)	35 (87.5%)	50 (84.7%)	
Double purse-string suture	3 (9.1%)	2 (6.3%)	1 (2.5%)	4 (6.8%)	
Manual	6 (18.2%)	5 (15.6%)	4 (10.0%)	5 (8.5%)	
Intraoperative blood loss > 500 mL	1 (3.0%)	2 (3.1%)	3 (7.5%)	1 (1.7%)	0.348
Number of positive lymph nodes	21.8 ± 7.6	21.9 ± 7.7	22.6 ± 8.0	24.7 ± 8.2	0.219
Distal resection margin < 1 mm	2 (6.1%)	1 (3.1%)	2 (5.0%)	3 (5.1%)	0.902

SD—standard deviation; IMA—inferior mesenteric artery.

**Table 4:** Post-operative features and outcomes of patients with rectal cancer during the period 2020–2022 in comparison with the pre-pandemic year 2019.

Variables	Control—2019 (n = 33)	2020 (n = 32)	2021 (n = 40)	2022 (n = 59)	*p*-Value
Biological response to radiation therapy	3 (9.1%)	4 (12.5%)	4 (10.0%)	7 (11.9%)	0.938
GCS < 15	3 (9.1%)	2 (6.3%)	4 (10.0%)	5 (8.5%)	0.849
Respiratory rate > 22	6 (18.2%)	5 (15.6%)	7 (17.5%)	9 (15.3%)	0.953
Temperature < 36 or >38	7 (21.2%)	6 (18.8%)	10 (25.0%)	11 (18.6%)	0.712
Heart rate > 100	9 (27.3%)	8 (25.0%)	13 (32.5%)	13 (22.0%)	0.502
Systolic BP < 100 mmHg	6 (18.2%)	5 (15.6%)	8 (20.0%)	10 (16.9%)	0.877
Pressor support	2 (6.1%)	2 (6.3%)	6 (15.0%)	4 (6.8%)	0.306
Mechanical ventilation	4 (12.1%)	3 (9.4%)	5 (12.5%)	7 (11.9%)	0.909
Peritoneal contamination	3 (9.1%)	4 (12.5%)	4 (10.0%)	5 (8.5%)	0.828
Ischemia of resection margins	2 (6.1%)	1 (3.1%)	2 (5.0%)	2 (3.4%)	0.290
Reintervention	2 (6.1%)	3 (9.4%)	3 (7.5%)	2 (3.4%)	0.475
Clavien–Dindo score					0.704
I	8 (24.2%)	7 (21.9%)	6 (15.0%)	19 (32.2%)	
II	11 (33.3%)	10 (31.3%)	9 (22.5%)	22 (37.3%)	
III	8 (24.2%)	9 (28.1%)	17 (42.5%)	12 (20.3%)	
IV	4 (12.1%)	4 (12.5%)	5 (12.5%)	4 (6.8%)	
V	2 (6.1%)	2 (6.3%)	3 (7.5%)	2 (3.4%)	
ICU admissions	4 (12.1%)	5 (15.6%)	6 (15.0%)	4 (6.8%)	0.314
Mortality rate	2 (6.1%)	3 (9.4%)	3 (7.5%)	2 (3.4%)	0.474

GCS—Glasgow Coma Scale; BP—blood pressure; ICU—intensive care unit.

## Data Availability

Data available on request.
